# Is the patient activation measure associated with adherence to colonoscopy after a positive fecal occult blood test result?

**DOI:** 10.1186/s13584-018-0270-8

**Published:** 2018-12-21

**Authors:** Revital Azulay, Liora Valinsky, Fabienne Hershkowitz, Racheli Magnezi

**Affiliations:** 1 Department of Management, MHA Program, Bar Ilan University, Ramat Gan, Israel; 2 Central Laboratory, Meuhedet Health Care, 5 Pesach lev, Lod, Israel; 3 Quality Department, Meuhedet Health Care, 124 Eben Gvirol, Tel Aviv, Israel

**Keywords:** Fecal occult blood test, Positive colorectal cancer screening, Colonoscopy, Adherence, Patient activation measurement, PAM

## Abstract

**Background:**

Colorectal cancer (CRC) is a major cause of morbidity and mortality worldwide, but these can be reduced significantly with population screening using annual fecal occult blood tests (FOBT)A positive FOBT requires timely follow-up with colonoscopy to maximize screening benefits.. Several barriers to follow-up have been identified, with patient health behaviors and choices comprising a significant part of these. The Patient Activation Measure (PAM) assesses knowledge, skills, beliefs, and confidence in managing health. Increased patient activation is related to positive health outcomes. The aim of this study is to examine the association between patient empowerment, as reflected in the PAM, and follow-up colonoscopy within 90 days of a positive FOBT result.

**Methods:**

This case-control study included 429 patients with a positive FOBT, 174 who had a colonoscopy within 90 days, and 255 who did not.. Participants completed a PAM telephone questionnaire (Cronbach’s α = 0.785). We used both univariate and multivariate analyses to examine the effect of the PAM score as on the likelihood of undergoing colonoscopy, after adjusting for the independent variables.

**Results:**

In this study we did not find a significant association between PAM and adherence to colonoscopy, using both univariate and multivariate analyses (*p* = .334 and *p* = .697, whether PAM was defined as a continuous or as categorical, respectively).

**Conclusions:**

This study was the first to examine the association between patient empowerment, as reflected in the patient activation measure, and adherence to colonoscopy after a positive FOBT. The findings did not support such an association. Further examination is required to clarify the relation between patient empowerment and activation and personal healthcare in general, and in the Israeli population in particular. Future policy should include specific, technical interventions to improve FOBT follow-up among all groups, until the patient-related barriers are better understood.

**Trial registration:**

ClinicalTrials.gov Identifier: NCT02534142 https://clinicaltrials.gov/ct2/show/NCT02534142

## Introduction

Colorectal cancer (CRC) is a major cause of morbidity and mortality throughout the world. In Israel, it is the second leading cause of cancer and cancer-related mortality [1]. When the disease is identified early and immediate surgical intervention is undertaken, survival is estimated at 90%. In Israel, for individuals ages 50–75 years, at average risk, the screening policy is an annual fecal occult blood test (FOBT), with Fecal Immunology test (FIT) [2].

A positive FOBT test result requires immediate follow-up with colonoscopy and surgical treatment to maximize screening benefits. Delay in follow-up after a positive FOBT can significantly undermine the benefits of CRC screening. Delays can increase CRC incidence, mortality and the net costs of screening, and reduce the number of life-years saved [3–6]. A large microsimulation model showed that every month of delay in follow-up after a positive FIT increases the mortality risk by 1.4% [5]. The recommended interval between a positive FOBT and a colonoscopy varies among countries, ranging from 30 to 180 days [7–13]. However, the proportion of individuals who undergo FOBT screening and do not continue with follow-up after a positive result range from 39 to 70% [7–10].

In Israel, the Ministry of Health (MOH) guidelines define a waiting period up to 90 days [14]. Follow-up rates after a positive FOBT in the Israeli population (over 1 million people) were 71%, and the median time to follow-up was 112 days; significantly longer than the recommended 90 days. Routine care following a positive FOBT result includes an automated computer alert that appears in the patient’s electronic medical record (EMR) to inform the patient’s physician. Physicians are expected to contact the patient regarding abnormal test results. Patients can also access their results on-line.

There have been several studies examining colonoscopy uptake for CRC. In general, adherence to colonoscopy recommendations is not high. In a study of 1455 patients who required a colonoscopy either for follow-up of previous findings or for screening, Johnson et al. [15] found that only a third of patients adhered to recommendations, with no difference between screening and follow-up. In a study of adherence to colonoscopy for first degree relatives of CRC patients, Bujanda et al. [16] found that of relatives who had been notified of their risk, only 38% underwent a colonoscopy. A similar study by Garcia et al. [17] found 51.8% adherence among first degree relatives of young patients with CRC.

In studies that examined socio-demographic variables and CRC screening behaviors, several factors have been identified as important. In a study by May et al. [18] ethnicity and socio-economic status were found to be significantly associated with screening colonoscopy: in general there was 50% adherence to recommendations, with African Americans and homeless patients having the lowest adherence rates (OR 0.37 and 0.53 respectively). Patients who take more prescription drugs, and those who do not have a primary health care provider also had lower uptake of CRC screening. Jibara et al. [19] examined the characteristics of 400 Hispanic Americans in relation to colonoscopy screening. Seventy percent reported receiving a recommendation from their physician to have a colonoscopy and of these 25% did not undergo the test. Younger patients and those not born in the US were significantly more likely to adhere. Fear of undergoing CRC screening was a strong predictor of non-adherence (*p* = 0.006).

Patient health status, knowledge, attitudes and health behavior have also been examined as factors in patient adherence to CRC recommendations. In a cross-sectional study of 378 patients with Inflammatory Bowel Disease [20], high self-efficacy was strongly correlated with colonoscopy adherence (OR 1.2. adjusted for health status and patient knowledge, *p* < 0.001). Hay et al. [21] examined the role of risk perception in promoting colorectal cancer (CRC) screening behavior in a diverse, inner city, primary care population. Perception of the chance of developing CRC was significantly associated with adherence to screening recommendations.

A cross-sectional study conducted in Israel by Bronner at al. [22] investigated the determinants of adherence to colonoscopy among 318 relatives of 164 patients with CRC. Investigators divided the study population into 3 groups- symptomatic screeners (who had a colonoscopy to investigate symptoms), asymptomatic screeners and non-screeners. Younger, Israeli-born, single and low income relatives of CRC patients were significantly less likely to have undergone a colonoscopy. Higher educational attainment and higher self-efficacy were significantly associated with adherence.

Awareness that patients should be active, effective managers of their own health and health care is growing. Hibbard et al. (2005) [23] developed the Patient Activation Measure (PAM) questionnaire to measure an individual’s ability to take control of his/her own healthcare. Increased patient activation, as measured with the PAM, was related to positive health outcomes [24, 25]. The PAM assesses knowledge, skills, beliefs, and confidence in managing health on a scale of four levels of activation. The lowest level includes passive recipients of care who do not believe in the need for active patient participation. The second level includes individuals who lack the confidence and/or knowledge to take health action. The third level includes individuals who are beginning to engage in recommended health behaviors. The fourth and highest level includes individuals who are proactive concerning their health. The PAM was previously investigated for efficacy among patients with chronic conditions, including HIV, and mental health problems [26–32].

As far as we know, the only study so far that examined the role of patient activation in CRC screening is Greene et al. [33]. In this study, routinely collected PAM scores from 25,047 EMR’s were used to examine associations between patient activation, health behaviors and CRC screening. The PAM was found to be significantly higher in high income areas, and was positively associated with 12 out of 13 health related outcomes, such as blood pressure and cancer screening, both as 4 levels and as a continuous score. Although the differences in CRC screening were not large (64.0% in the lowest PAM group up to 67.4% in the highest), they were statistically significant. In a multivariate model, the PAM score was significantly associated with CRC screening for the lowest and highest socio-economic levels, but not the middle level.

The current study is the second study that examines the association between CRC screening and PAM, and the first to examine the association between patient activation levels and adherence to a follow-up colonoscopy after a positive FOBT. Identifying a link between activation and follow-up will enable us to find ways to increase adherence through targeted interventions.

## Methods

This study was conducted in Meuhedet, one of four health maintenance organizations (HMO) in Israel. Meuhedet insures and provides care for 1.2 million members. The current rate of colorectal screening is 60% among 180,000 Meuhedet members ages 50–75 years, similar to the rate of the entire Israeli population [14]. In Meuhedet from an unpublished data, the rate of follow-up colonoscopy after a positive FOBT was 41%, which is extremely low.

This study was approved by the Meuhedet *Institutional Review Board* on May 20, 2015 (IRB; trial reference number: 02–20–05-15).

ClinicalTrials.gov Identifier: NCT02534142.

https://clinicaltrials.gov/ct2/show/NCT02534142

### Study population

This retrospective cross-sectional study was conducted in 2016. The study population consisted of all Meuhedet members, ages 50–75 years, who had a positive FOBT in 2015. Members were excluded if they: (1) had a personal or family history of CRC (as the recommended test for them is colonoscopy and not FOBT), (2) had undergone colonoscopy 10 years before the positive FOBT (colonoscopy screening is recommended every 10 years, and further screening tests are not required during this period), (3) were diagnosed with any type of cancer during the study period, and (4) Physicians did not approve contacting the patient (for each patient we received approval to contact them from their primary physician as the IRB required. The reasons were mainly technical (patient residing abroad or medical reasons such as patients with dementia). Of 1369 patients who were approved by their physicians, 732 (53.5%) could not be reached over the phone (at least three separate attempts, to both landlines and mobile telephones), 176 (12.9%) refused to participate, and 32 (2.3%) had family history of CRC. The final study sample consisted of 429 people. No substantive distribution differences were found neither for gender, for age or for SES between participants and non-participants (55.0% males in participants, vs. 53.2%, *p* = 0.32), mean age 62 in both groups (62.11 + − 6.65 in participants, vs. 61.72 + − 7.0, *p* = 0.242), and mean SES 10 in both groups) (9.96 + − 3.66 in participants, vs. 9.67 + − 3.21, *p* < 0.014).

Patients were considered to be adherent if they completed a colonoscopy up to 90 days from the positive FOBT. We used a 90-day threshold as this is the standard used by the Israeli Ministry of Health, and 93% of colonoscopies were completed before the 90-day period. Among those defined as “non-adherent” (did not have a colonoscopy within 90 days), 4.2% had a colonoscopy within between 91 and 120 days, and 2.6% between 121 and 154 days. Overall they comprised 5.09% of the “non-adherent” population.

As the aim of this study was to examine the association between patient activation and CRC follow-up, all study participants were contacted by telephone and interviewed using the Patient Activation Measure (PAM) questionnaire 12–18 months following the FOBT. A short, 13-item version of the PAM questionnaire was validated in 2005 [23]. As required by the IRB, when a patient reported he/she had not undergone follow-up colonoscopy, the interviewer advised them to contact their physician regarding the FOBT results.

### Study variables

Independent Variables were PAM score, gender, age, marital status, ethnicity, country of birth and SES. SES is derived from the member’s home address, and based on the Israeli Census Bureau (ICB) locality definitions [34]. SES levels range from 1 to 20; 20 is the highest. We had SES data for 97.4% of participants, and missing values were due to participants living in new neighborhoods or those not defined in the ICB database. For the purpose of this study, SES levels were grouped into three levels: 1–7 low, 8–13 intermediate, and 14–20 high. Although SES is sometimes divided into 4 categories, due to the relatively homogenous nature of our population this would have resulted in cells with less than 10% and impacted negatively on the stability of statistical analysis, and this is an accepted method of categorizing the population [14]. We also examined health status and risk factors that obtained from the EMR, such as smoking (as documented in the EMR by the physician. As a rule, a smoker is defined as someone who smokes at least once a day. We did not validate the accuracy of this information, but relied on the physician’s documentation), overweight and obesity (Overweight = 25.1–30; Obese> 30), hypertension (Systolic Blood Pressure (SBP) > 130 mmHg; Diastolic Blood Pressure (DBP) > 90 mmHg), cholesterolemia (LDL Cholesterol> 100 mg/dl) and medication adherence. Medication adherence was obtained from the EMR, and was defined as at least 9 purchases within the last 12 months of medications used for hyperlipidemia and cholesterolemia.

The dependent variable was adherence to colonoscopy within 90 days after a positive FOBT.

### Data sources

Positive FOBT results were retrieved from the Meuhedet Central Laboratory database. Patient demographic and clinical characteristics were obtained from the EMR.

### PAM questionnaire

A previously-validated Hebrew version of the PAM [35] was used in this study. Responses were rated on a Likert Scale of 1 to 5, according to the degree of agreement with health management statements; e.g., “I know how to prevent problems with my health” or “I am confident that I can tell a doctor my concerns, even when he or she does not ask”. The five response categories were: (1) strongly disagree, (2) disagree, (3) agree, (4) strongly agree, and (5) not applicable. The mean scores were transformed to a PAM score ranging from 0 to 100, based on scoring rules of Insignia Health [36]. Based on the same rules, the PAM score was also converted into four levels of patient activation. To facilitate interpretation, patient activation was described as level 1 (passive) to level 4 (proactive). The questionnaire used for this study was validated in English, Hebrew, Arabic and Russian. In our study, participants were offered the option of any of these languages.

### Statistical analysis

Descriptive statistics were used to summarize demographic characteristics. Cronbach’s alpha was used to calculate internal consistency between the 13 items on the PAM questionnaire.

Chi-square test was used to examine the association between discrete variables. t-test and one-way ANOVA were used to compare means of continuous variables. Logistic regression was used to ascertain the effect of the PAM score as a continuous variable on the likelihood of undergoing colonoscopy, after adjusting for confounding variables found to be associated with the dependent variable.

Data were analyzed with IBM SPSS Statistics for Windows, Version 24.0. (Armonk, NY). *P*-values < 0.05 were considered significant for all analyses.

## Results

Characteristics of study population by adherence to colonoscopy within 90 days after a positive FOBT are presented in Table 1. A total of 429 eligible patients were included, with a mean (±SD) age of 62.11 ± 6.65 years, mean (±SD) SES level of 9.96 ± 3.66 and mean (±SD) PAM of 62.07 ± 16.55. There were 255 participants (59.4%) in the non-adherent group and 174 (40.6%) in the adherent group.

**Table 1:** Characteristics of study population by colonoscopy adherence

Characteristic	N(%)	Colonoscopy		*p*-value
		174 (40.6%)	255 (59.4%)	
		Yes (N, %)	No (N, %)	
Gender				
Male	236 (55.0)	93 (53.4)	143 (56.1)	0.622
Female	193 (45.0)	81 (46.6)	112 (43.9)	
Age group				
Mean(SD)	62.11 (6.65)	62.2 (6.63)	61.96 (6.68)	0.578
50–54	78 (18.2)	29 (16.7)	49 (19.2)	0.43
55–59	76 (17.7)	28 (16.1)	48 (18.8)	
60–64	104 (24.2)	46 (26.4)	58 (22.7)	
65–69	114 (26.6)	52 (29.9)	62 (24.3)	
70–75	57 (13.3)	19 (10.9)	38 (15.0)	
Marital Status				
Single, Divorced or Widowed	116 (27.0)	45 (25.9)	71 (27.8)	0.65
Married or living with a partner	313 (73.0)	129 (74.1)	184 (72.2)	
Country of Birth				
Israel	163 (38.0)	59 (33.9)	104 (40.8)	0.15
Other	266 (62.0)	115 (66.1)	151 (59.2)	
Ethnicity				
Jewish	344 (80.2)	135 (77.6)	209 (82.0)	0.264
Non-Jewish	85 (19.8)	39 (22.4)	46 (18.0)	
Socioeconomic status (*n* = 418, 11 missing)				
Mean (SD)	9.96 (3.66)	9.66 (3.59)	9.95 (3.72)	0.985
Low 1–8	115 (27.5)	46 (27.1)	69 (27.8)	0.649
Intermediate 9–13	239 (57.2)	101 (59.4)	138 (55.6)	
High 14–20	64 (15.3)	23 (13.5)	41 (16.6)	
Smoking (*n* = 385, 44 missing)				
yes	70 (18.2)	21 (13.7)	49 (21.1)	0.066
no	315 (81.8)	132 (86.3)	183 (78.9)	
BMI (*n* = 423, 6 missing)				
normal	100 (23.6)	34 (19.8)	66 (26.2)	0.118
overweight	162 (38.3)	75 (43.9)	87 (34.5)	
obese	161 (38.1)	62 (36.3)	99 (39.3)	
Adherence to cholesterol medication among 233 patients with hypercholesterolemia				
less than 9 purchases a year	115 (49.4)	48 (51.6)	67 (47.9)	0.574
9+ purchases	118 (50.6)	45 (48.4)	73 (52.1)	
Adherence to hypertension medication among 212 patients with high blood pressure				
less than 9 purchases a year	45 (21.2)	14 (16.7)	31 (24.2)	0.188
9+ purchases	167 (78.8)	70 (83.3)	97 (75.8)	
PAM continuous				
Mean(SD)	62.07 (16.55)	62.77 (16.56)	61.59 (16.55)	0.472
PAM categorical				
Level 1- passive receipt of care	93(21.6)	37 (21.2)	56 (21.9)	0.774
Level 2	71(16.6)	28 (16.1)	43 (16.9)	
Level 3	87(20.3)	32 (18.4)	55 (21.6)	
Level 4- proactive self-care	178(41.5)	77 (44.3)	101 (39.6)	

One quarter (23.7%) of the sample completed the questionnaire in Russian. Although there were Arabic speakers in our population, the level of their spoken Hebrew was such that they preferred to be interviewed in Hebrew. There was no difference in PAM levels between participants in the two languages (*p* = 0.090).

In terms of demographic variables, the study population was 55.0% male, approximately half the participants belonged to age groups 60–64 and 65–69 (24.2 and 26.6%, respectively). Most were married or lived with a partner (*N* = 313, 73.0%), were born outside of Israel (*N* = 266, 62.0%), were Jewish (344, 80.2%) and were in the intermediate SES level 9–13 (239, 57.2%). In terms of health-related variables, most were non-smokers (81.8%), adhered to cholesterol medication recommendations (50.6%), adhered to hypertension medication recommendations (78.8%) and were obese (38.1%) and overweight (38.3%). The characteristics of those who adhere and those who did not adhere to follow-up colonoscopy referral are presented in Table 1. Although there were some differences between subgroups, none are statistically significant.

Patients were also evaluated according to the independent variables to determine differences in mean PAM scores. The 13 PAM items showed high internal reliability (Cronbach’s alpha = 0.785). The PAM score (mean = 62.07 ± 16.55) was divided into four categories, according to the PAM questionnaire protocol. In this study, 41.5% patients were in the highest level 4 (proactive self-care), and 21.6% were in the lowest level 1 (passive receipt of care) (Table 1). Univariate analysis didn’t reveal any significant association between PAM and adherence to colonoscopy, whether PAM was defined as a continuous (*p* = .472) or as a categorical (*p* = .774) variable. The percentage of patients who had a PAM level 4 was higher among males compared with females (42.8% vs. 39.9%, respectively, *p* = .090), among native born Israelis as compared with those born outside of Israel (49.1% vs. 36.8%, respectively, *p* = .033). For age, marital status, ethnicity and SES level no significant association with PAM was shown.

Since no variable was found to be related to adherence, we performed a multivariable logistic model that included only the PAM study variable, once as a continuous variable and once as categorical variable, and age and gender, as universal variables. This model revealed no significant associations between PAM and adherence to colonoscopy. To test the robustness of our findings, we conducted a number of analyses, including a model with all the covariates presented earlier and several models with the PAM variable, age and SES as categorical covariates. In all cases, there was no significant correlation between the variables, and specifically – a multivariate model using PAM as a categorical variable yielded results that were very similar to the model presented in Table 2. In addition, to test whether the observed relationships between patient activation and the health-related outcomes were consistent for those of different SES, we tested for significance of interaction terms between patient activation and income tertile. The interaction was consistently not significant and did not alter the model main effects. We finally decided to present a model that includes the more reliable and stable variables in our database and include fewer missing values (Table 2). In all the tests performed, no covariate was found to have any significant independent effect on the dependent variable. The area under the ROC curve (AUC) was 0.567 (*p* < .05). Although the authors of the original PAM test did not specify subdivision of the items into scales, it is possible to distinguish between items relating more to knowledge and responsibility as opposed to items relating more to health control and management. Therefore, we also performed regression with those subscales as predictors. No associations between PAM and adherence were demonstrated (*p* = 0.697). We repeated the regression using all patients who had a colonoscopy, regardless of the time to colonoscopy (*n* = 187), and the results were the same.

**Table 2:** Multivariate logistic model of the effect of PAM on the likelihood of undergoing follow-up colonoscopy (*N* = 429)

Variable	Odds ratio	95% Confidence Interval		*p*-value
		Lower	Upper	
Mean PAM score (continuous)	1.006	0.994	1.018	0.334
Gender: female vs. male	1.175	0.786	1.759	0.432
Ethnicity: Non-Jewish vs. Jewish	1.380	0.835	2282	0.209
Native born Israeli vs. immigrant	0.702	0.451	1.094	0.118
Marital status: live alone vs. living with a partner	0.851	0.541	1.341	0.488
Age (continuous)	1.000	0.969	1.033	0.978
SES (continuous)	1.005	0.950	1.063	0.862
Constant	0.453			0.468

## Discussion and conclusion

This study aimed to examine the association between patient empowerment as expressed by patient activation levels and adherence to follow-up after a positive FOBT. Patient activation is one of the measures that illustrate the degree to which patients take control of their own healthcare.

The aim of this study was to examine associations between the PAM levels and CRC follow-up. The distribution of PAM levels in our population is similar to that found in other studies [23, 33]. In this study, we did not find significant correlations between PAM and age, ethnicity, SES and marital status. These findings were similar to those described by Mazanec et al., who focused on patients with CRC [37]. Most of the study population in Mazanec study reported levels 3 or 4 of activation and were stable over time, but not significantly correlated with any of the independent variables, including ethnicity, employment, education status, relationship to the care recipient, or cancer stage. We found no significant correlation between PAM levels and health status and risk factors such as – smoking, obesity, hypertension, hypercholesterolemia and adherence to medications related to these conditions. This is unlike recent studies that confirmed that patients’ activation significantly affects their reported medication adherence [38] and that patient activation seems to be an important and modifiable factor for influencing chronic disease outcomes [39].

Patient activation was similar among Jews and non-Jews, but was significantly higher among those native born Israelis than among immigrants. One potential explanation for this finding is that immigrant’s encounter a language barrier do not understand the test result and what is expected to do next or have difficulties navigating the health care system. Another explanation is that immigrants have different culture behaviors and beliefs that can be a barrier to follow up [40].

Previous studies that examined PAM in patients with chronic conditions [30, 33, 37] demonstrated that those with high PAM scores were significantly more likely to display self-management behavior, use self-management services, and report high medication adherence, as compared with patients with the lowest PAM scores. One potential explanation for the different findings in the present study was that, in this instance, adherence to follow-up was influenced by extrinsic factors, such as access or physician influence, more than intrinsic factors, particularly because the follow-up activity was highly medically related [41–43]. There is a lack of consensus among physicians regarding the value of a fecal occult blood test as a screening tool for CRC [44]. Several studies have shown that a combination of personal and extrinsic factors influence patients’ decisions and behavior regarding screening. Extrinsic factors include physician attitudes, [15, 17], and when a negative attitude is combined with a lack of awareness and knowledge among patients [45] the result may be conflicting messages regarding the test and the follow-up required, and this may reduce the adherence rate regardless of patient activation.

We did not find significant association between PAM and adherence to colonoscopy. It is possible that a personal barrier to a colonoscopy had more impact than the traits demonstrated by the PAM score.

Having a colonoscopy consists of several technical steps – obtaining a physician referral, scheduling an appointment, undergoing the required preparation and arriving at the clinic. It also involves undergoing some psychological adjustment. First, most people who have a screening test, such as a fecal occult blood test, expect the results to be negative, and are startled when they are not. Second, the actual colonoscopy is an invasive procedure, and may be frightening. Additionally, the preparation is physically difficult, and therefore conceptually intimidating. Most importantly, managing a chronic disease is a continuous process over a long period of time, and is essentially different both emotionally and cognitively, from a positive screening test follow-up procedure. It is possible that if the initial period after a chronic disease diagnosis were investigated, the findings would be similar to those in our study [27, 29, 30].

There are a number of important studies that examined the association between colonoscopy performance and behavioral elements. In a study of 342 first degree relatives of patients with CRC [16] the only personal variable associated with adherence was that the relative with CRC was young. This may reflect a higher perception of risk. Friedman et al. [20] found that high self-efficacy among symptomatic patients with irritable bowel disease was associated with higher adherence to surveillance colonoscopy, but perception of risk was not. This is similar to findings by Bronner et al. [22] who showed that self-efficacy was associated with adherence, whereas patients who did not have screening colonoscopies tended to emphasize the reasons to abstain, such as fear and anxiety or embarrassment. In view of these studies, although it is surprising that PAM- which measures patient activation, that is not very different from self-efficacy was not associated with colonoscopy follow-up, perhaps the negative aspects of colonoscopy such as expected discomfort and anxiety were important influences on the decision to complete follow-up. In the study by May et al. [18] lower service connectedness, and not having a primary care physician – both related to health behaviors – were associated with lower screening colonoscopy rates. Overall, patients who are more connected to their healthcare provider, perceive the risk to be higher, have more knowledge about the test and have higher self-efficacy are more likely to adhere to screening colonoscopy recommendations. The PAM may measure some of these attributes as components of patient activation, we expected to find an association between adherence and PAM. As we did not measure any of these separately, we cannot ascertain whether our findings reflect variations between PAM and other measures of self-efficacy and knowledge, or whether follow-up after a positive FOBT is influenced by different factors.

The current study had several limitations. We used a cut-off time to colonoscopy of 90 days after the positive FOBT result. In a larger study in Meuhedet, during the years 2012–2015 the mean time to colonoscopy was 70.13 days, median 55.0, SD 62.56. In this study, few patients (7%) had a colonoscopy 91 days and more after the initial FOBT, but it was impossible to set a different cutoff point as the remaining colonoscopies were spread over a period of up to an additional year. Changing the cutoff period to 120 or 150 days did not change our findings, indicating that access barriers did not significantly impact follow-up. Another limitation of this study was that SES was not measured directly – it was obtained from the SES of the participants home address area based on postal codes. It is possible that an individual with high income or education levels resides in a low SES address. However, this methodology is common in research conducted as part of the Community Quality Indicator program in the Israeli Ministry of Health [14]. Another potential limitation is the time gap between the FOBT result and the timing of the telephone interview, as patient activation may vary with time and context. It is possible that had we measured PAM at the time of the result, different levels would have been observed. A potential bias is the lack of information regarding the proportion of those born outside of Israel in the population of Meuhedet in these age groups. It might indicate that our study group may have potential bias regarding the country of birth, in light of the low response rate. Over half of the study population could not be reached using the numbers provided to Meuhedet (at least three attempts). It is possible that this population differs from the final population as their availability is lower, although this is unlikely as most had mobile telephones, and this was probably a matter of the timing of the telephone calls, and the groups were similar in age, gender and SES. Another limitation is that we did not validate the PAM questionnaire results with other associated behavioral variables, such as fear of the test, self-efficacy and risk perception, that may have been useful in explaining our findings.

## Conclusion

This study was the first to evaluate whether there is an association between patient empowerment, as reflected in the patient activation measure, and adherence to colonoscopy after a positive FOBT. Our findings did not support such an association. Further examination is required to clarify the relation between patient empowerment and activation and personal healthcare in general, and in the Israeli population in particular. These findings presented here contribute to the growing literature on patient activation measures and health behaviors. Future policy should include specific, technical interventions, such as text reminders, to improve FOBT follow-up among all groups, until the patient-related barriers are better understood.

## Abbreviations

AUC: Area Under Curve
CRC: Colorectal Cancer
DBP: Diastolic Blood Pressure
EMR: Electronic Medical Record
FIT: Fecal Immunology Test
FOBT: Fecal Occult Blood Test
HMO: Health Maintenance Organizations
ICB: Israeli Census Bureau
IRB: *Institutional Review Board*
MOH: Ministry of Health
PAM: Patient Activation Measure
SBP: Systolic Blood Pressure
SES: Socioeconomic status
